# Supplementary material for: Structural basis of antibody inhibition and chemokine activation of the human CC chemokine receptor 8
Source: Nat Commun. 2023 Dec 1;14:7940. doi: 10.1038/s41467-023-43601-8 (PMC10692165; doi:10.1038/s41467-023-43601-8)
Supplement: Supplementary file 1 — Supplementary Information [file 41467_2023_43601_MOESM1_ESM.pdf]

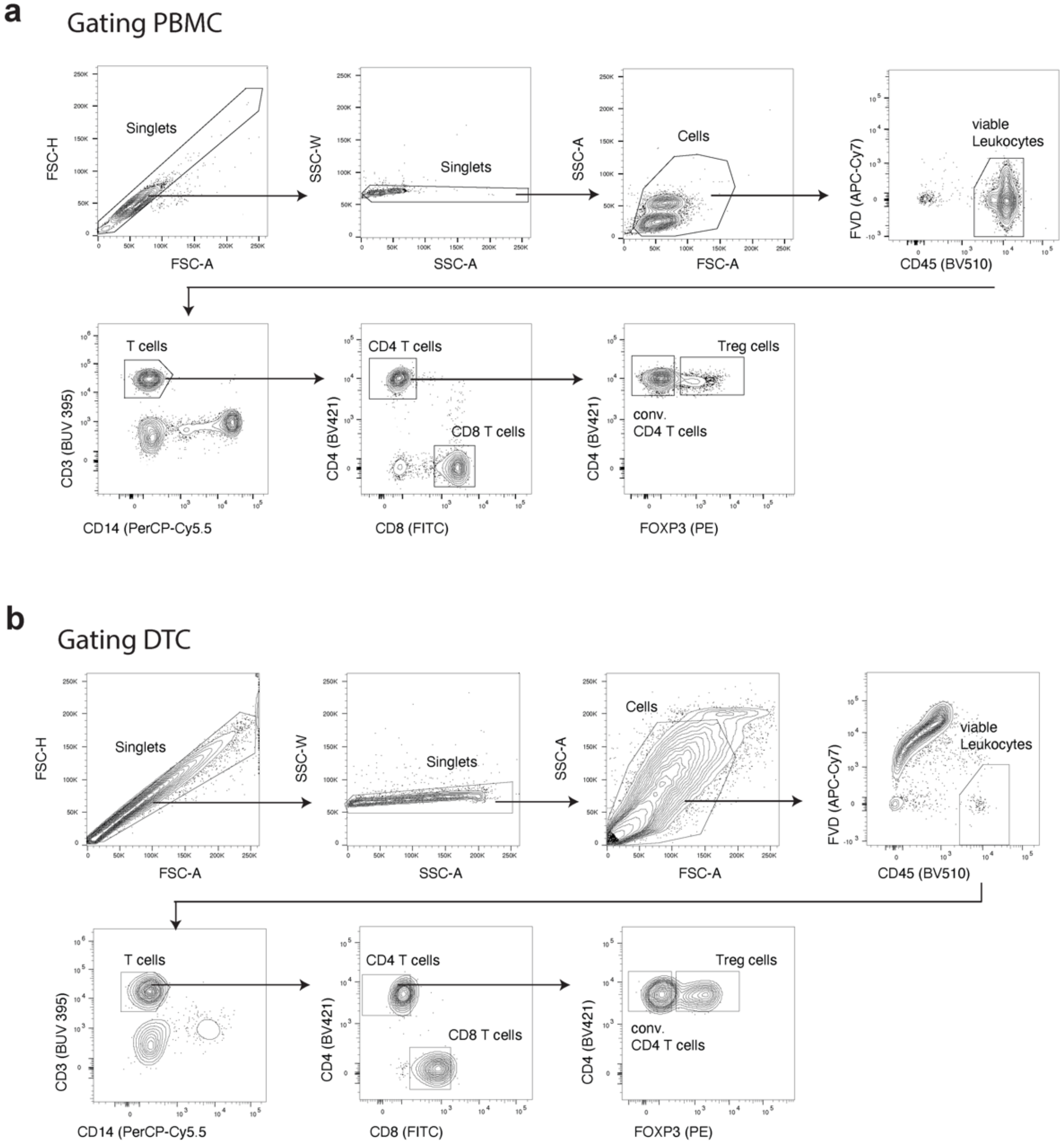

Supplementary Fig. 1: : Gating strategy for flow cytometry analysis of mAb1 and clone 433H binding to human PBMCs and DTCs.

a Human peripheral blood mononuclear cells (PBMC) or **b** dissociated tumor cells (DTC) were stained for CCR8 expression and several T cell lineage markers and analyzed by flow cytometry. **a, b** Contour plots outlining gating strategy used to identify Treg cells, CD8 T cells and CD4 conventional T cells.

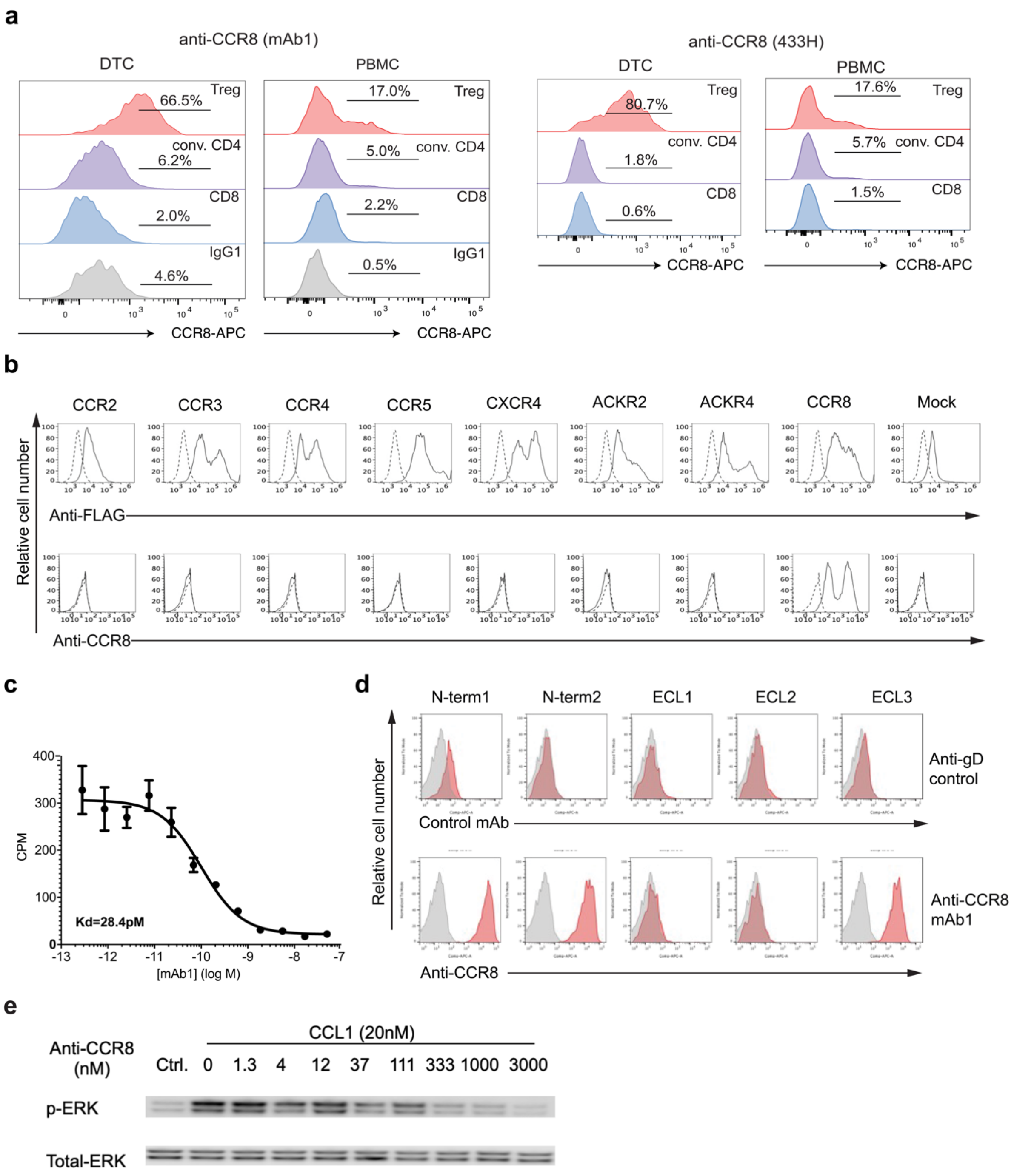

Supplementary Fig. 2: Characterization of mAb1 binding

a Flow cytometric analysis of anti-CCR8 antibodies mAb1 and 433H binding to subsets of human peripheral blood mononuclear cells (PBMCs) or dissociated tumor cells (DTCs). Histogram graphs showing anti-CCR8 binding to Treg cells, conventional CD4 T cells, and CD8 T cells compared to an isotype (IgG1) antibody. Numbers indicate frequencies of CCR8-expressing cell fraction. **b** Cell surface binding experiments comparing anti-Flag and mAb1 binding to HEK293 cells transiently transfected with FLAG-tagged chemokine receptors. MAb1 only bound to CCR8-expressing cells, while isotype control

anti-gD antibody did not bind to any of these GPCRs, thus demonstrating the selectivity of mAb1 for CCR8. **c** Scatchard analysis of mAb1 binding to CHO cells stably expressing human CCR8. Measurements were performed in triplicate. **d** Flow cytometry analysis of mAb1 binding to HEK293 cells transiently transfected with Flag-tagged CCR8.CCR5 chimeras, where individual extracellular regions of CCR8 were replaced with the corresponding region from CCR5. For **b** and **d**, cell surface expression of each GPCR construct was confirmed by staining with an anti-FLAG antibody control. The mAb1 binds to N-terminal and ECL3 chimeras, but not to ECL1 and ECL2 chimeras, indicating that the latter are essential for mAb1 recognition of CC8. **e** Analysis of mAb1-mediated inhibition of CCL1-induced ERK phosphorylation. huCCR8.Jurkat cells were incubated with anti-CCR8 antibody mAb1 for 30min, then 20nM CCL1 was added for 5min. Cell lysates were analyzed by western blotting using anti-phospho-ERK or anti-ERK antibody. Bands were quantified and pERK values were normalized to the corresponding total ERK for each condition, as shown in **Fig. 1c**. Data are representative of two independent experiments. Source data are provided as a Source Data file.

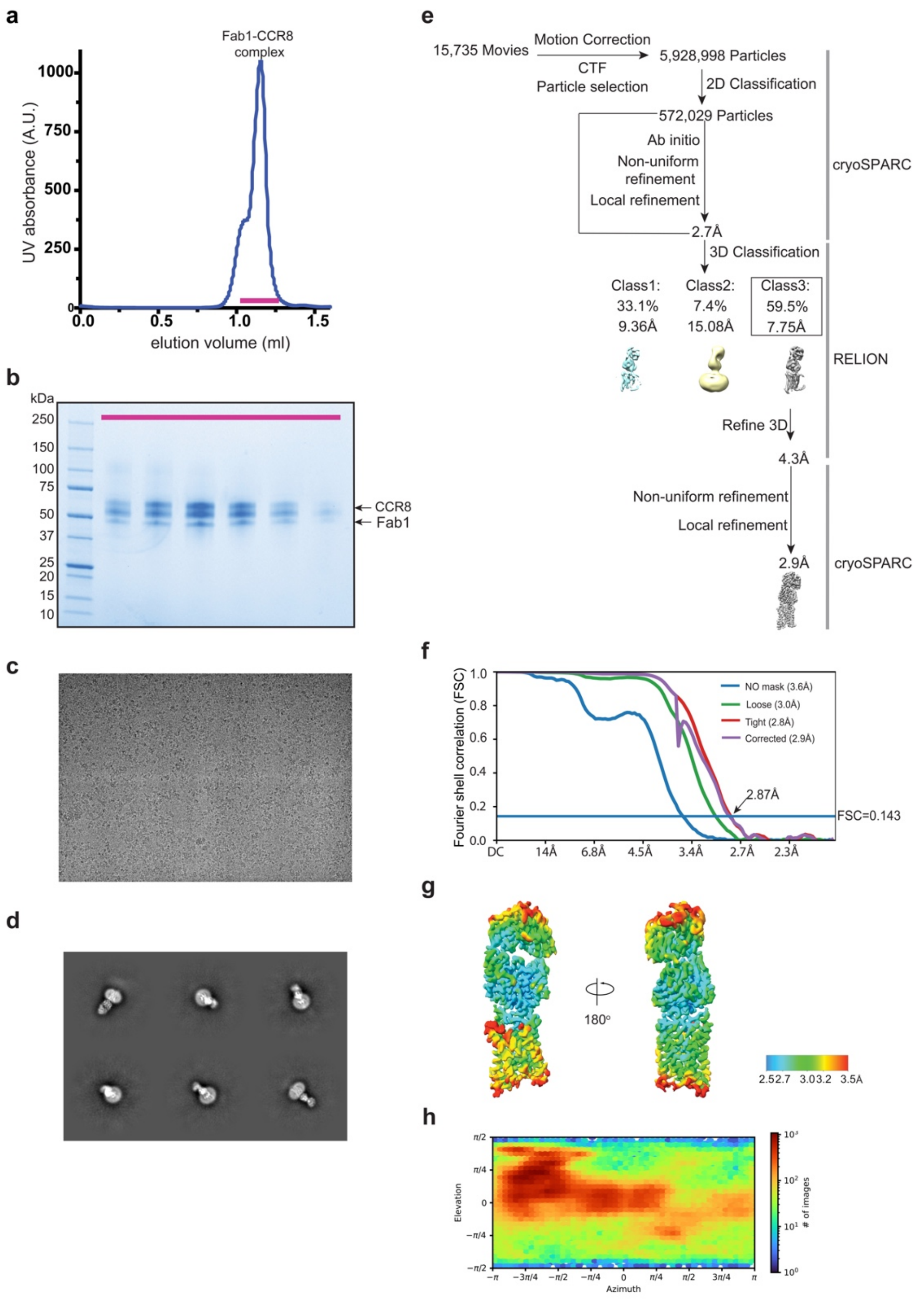

Supplementary Fig. 3: Purification and cryo-EM data processing of Fab1-CCR8 complex.

a-b Size-exclusion chromatography (SEC) elution profile (**a**) and SDS-PAGE analysis (**b**) of the Fab1-CCR8 complex. **c-d** Representative cryo-EM micrograph (**c**) and representative 2D class averages (**d**) of the Fab1-CCR8 complex. **e** Cryo-EM data processing workflow. **f** Gold-standard Fourier shell correlation (FSC) curve, indicating an overall resolution of 3.1 Å for the Fab1-CCR8 complex. **g** Cryo-EM density map colored by local resolution. **h** Angular distribution of particle projections for Fab1-CCR8 reconstruction.

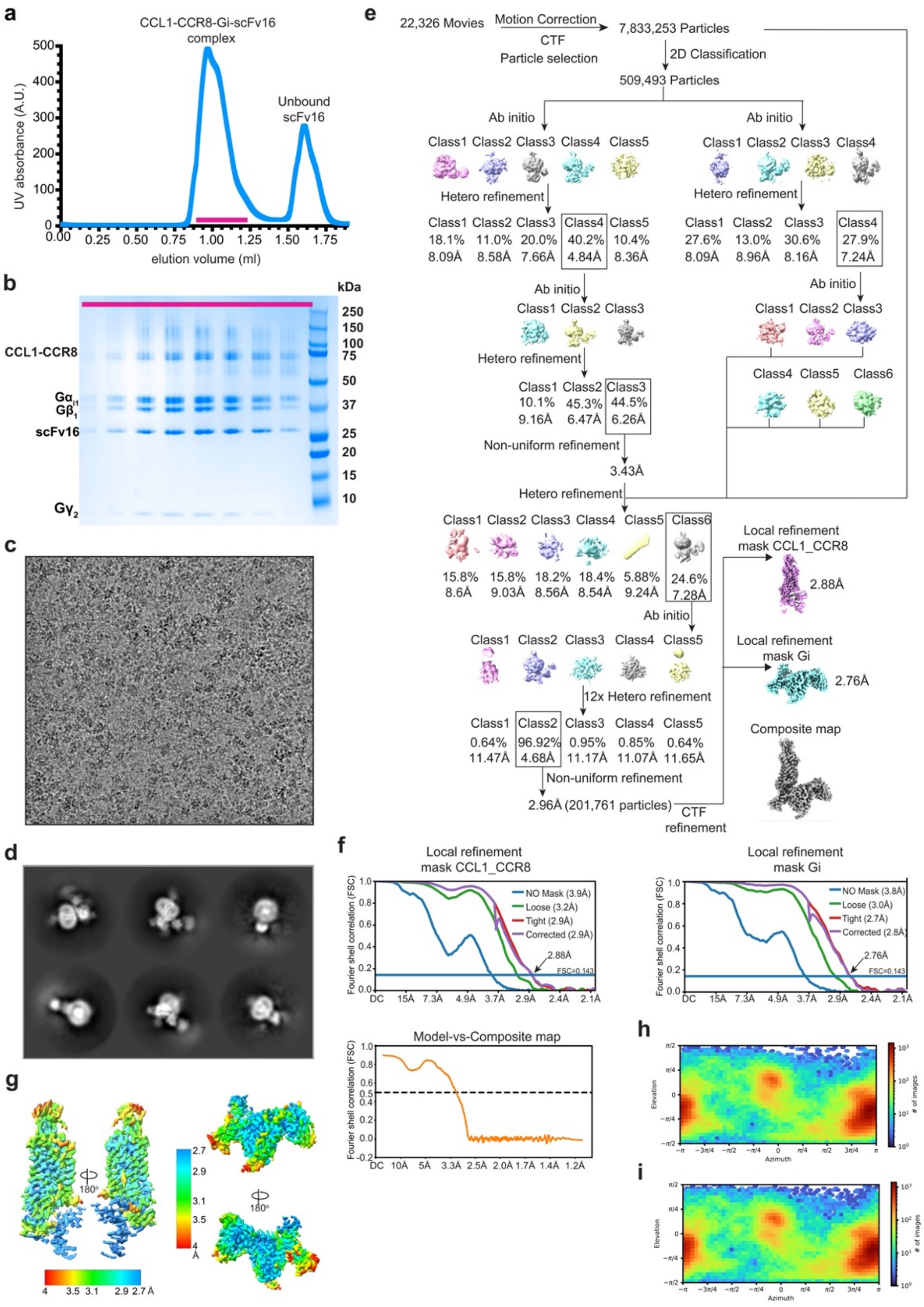

Supplementary Fig. 4: Purification and cryo-EM data processing of CCL1-CCR8-G$_i$-scFv16 complex.

a-b SEC elution profile (**a**) and SDS-PAGE analysis (**b**) of the CCL1-CCR8-G$_i$-scFv16 complex. **c-d** Representative cryo-EM micrograph (**c**) and representative 2D class averages (**d**) of the CCL1-CCR8-G$_i$-scFv16 complex. **e** Cryo-EM data processing workflow. **f** Gold-standard Fourier shell correlation (FSC)

curve, indicating an overall resolution of 2.9 Å for the CCL1-CCR8-G_i-scFv16 complex. **g** Cryo-EM density map colored by local resolution. **h-i** Angular distribution of particle projections for (**h**) CCL1_CCR8 and (**i**) Gi_scFv16 reconstruction.

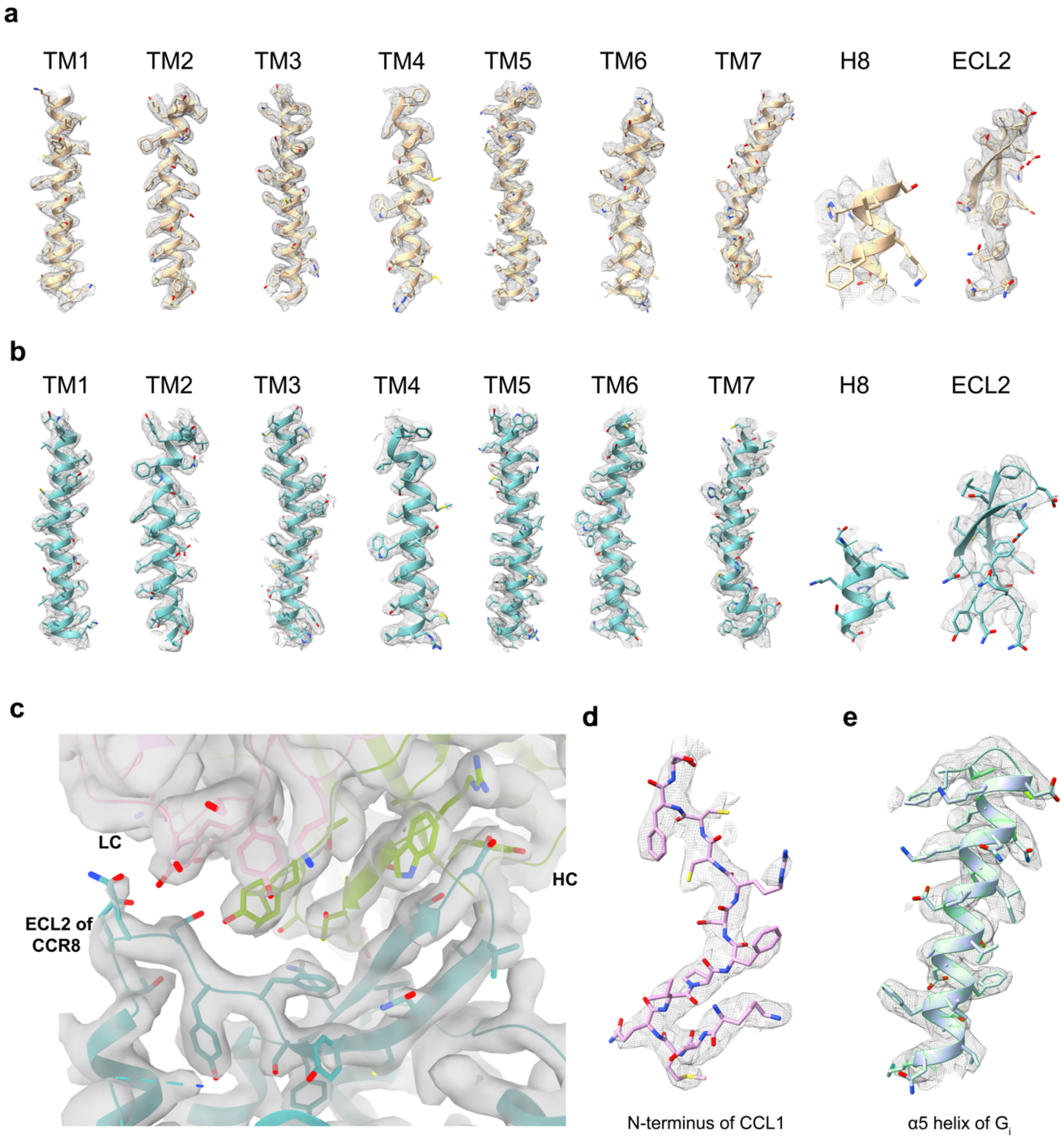

Supplementary Fig. 5: Electron density maps of the Fab1-CCR8 and CCL1-CCR8-G_i-scFv16 structures.

a-b Cryo-EM density map for all transmembrane helices and ECL2 of CCR8 in the Fab1-CCR8 (**a**) and the CCL1-CCR8-G_i-scFv16 structures (**b**). **c** Cryo-EM map of the interface between CCR8 and Fab1. **d** Cryo-EM density map of the CCL1 N-terminus. **e.** Cryo-EM density map of the alpha 5 helix of Galpha i.

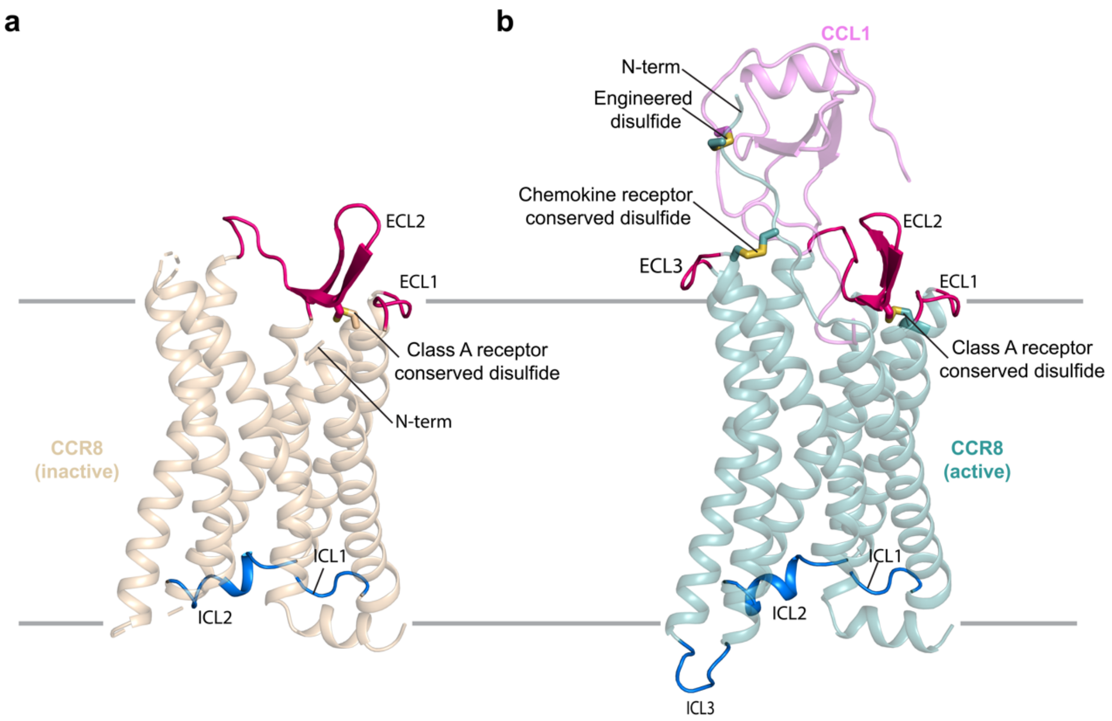

Supplementary Fig. 6: Comparison of extracellular regions in the Fab1- and CCL1-bound CCR8 structures

a-b Side-by-side comparison of the CCR8 structure in the Fab1-bound, inactive state (**a**) and in the CCL1-bound, active state (**b**). The extracellular and intracellular regions that are resolved in each structure are highlighted in red and blue, respectively. Disulfide bridges present in each CCR8 structure are shown as yellow sticks and labeled.

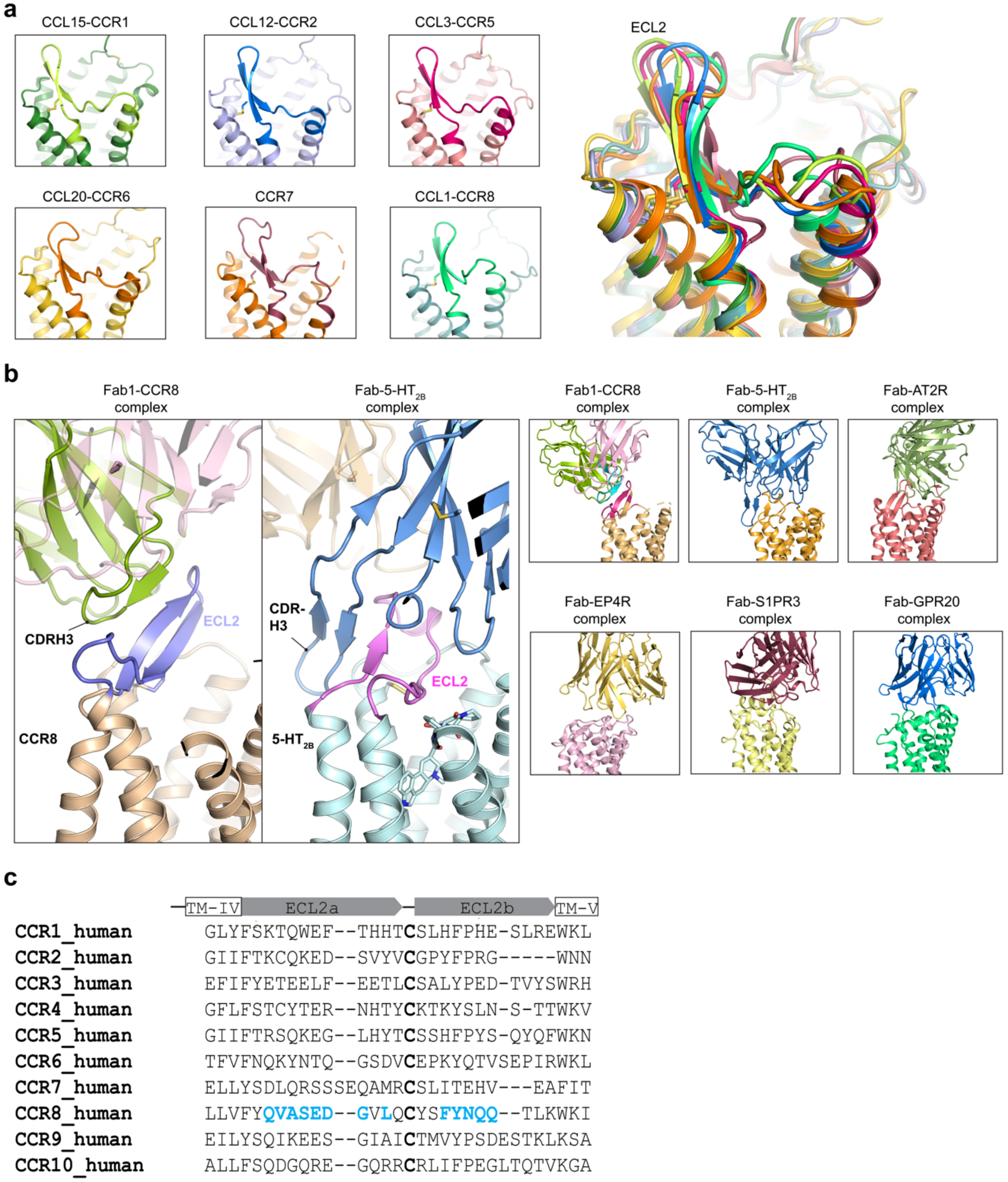

Supplementary Fig. 7: Comparison of the ECL2 fold across chemokine-GPCR and Fab-GPCR complexes

a Comparison of ECL2 in different C-C chemokine family receptors: CCR1-CCL15-G_i (PDB ID: 7VL9, green), CCR2-CCL12-G_i (PDB ID: 7XA3, blue), CCR5-CCL3-G_i (PDB ID: 7F1Q, pink), CCR6-CCL20-G_o (PDB ID: 6WWZ, orange), CCR7-Cmp2105 (PDB ID: 6QZH, red) and CCR8-CCL1-G_i are shown separately (left panels) and superimposed (right panel). **b** Comparison of the ECL2 conformation in representative structures of class A GPCRs in complex with Fabs engaging the receptor extracellular

loops (FabP2C2-HT_{2B}, PDB ID 5TUD; Fab-AT2R, PDB ID 5XJM; Fab-EP4R, PDB ID 5YWY; Fab-S1PR3, PDB ID 7C4S; Fab-GPR20, PDB ID 8HS2) to our Fab1-CCR8 structure. The FabP2C2 -bound 5-HT_{2B} structure (PDB ID: 5TUD) stands out as being the only one where the Fab also engages the ECL2 β-hairpin through antiparallel β-strand pairing with its CDRH3, similar to our Fab1-CCR8 structure. **c** Sequence alignment of ECL2 in human C-C chemokine family receptors. Residues in the CCR8 sequence interacting with Fab1 are highlighted in blue.

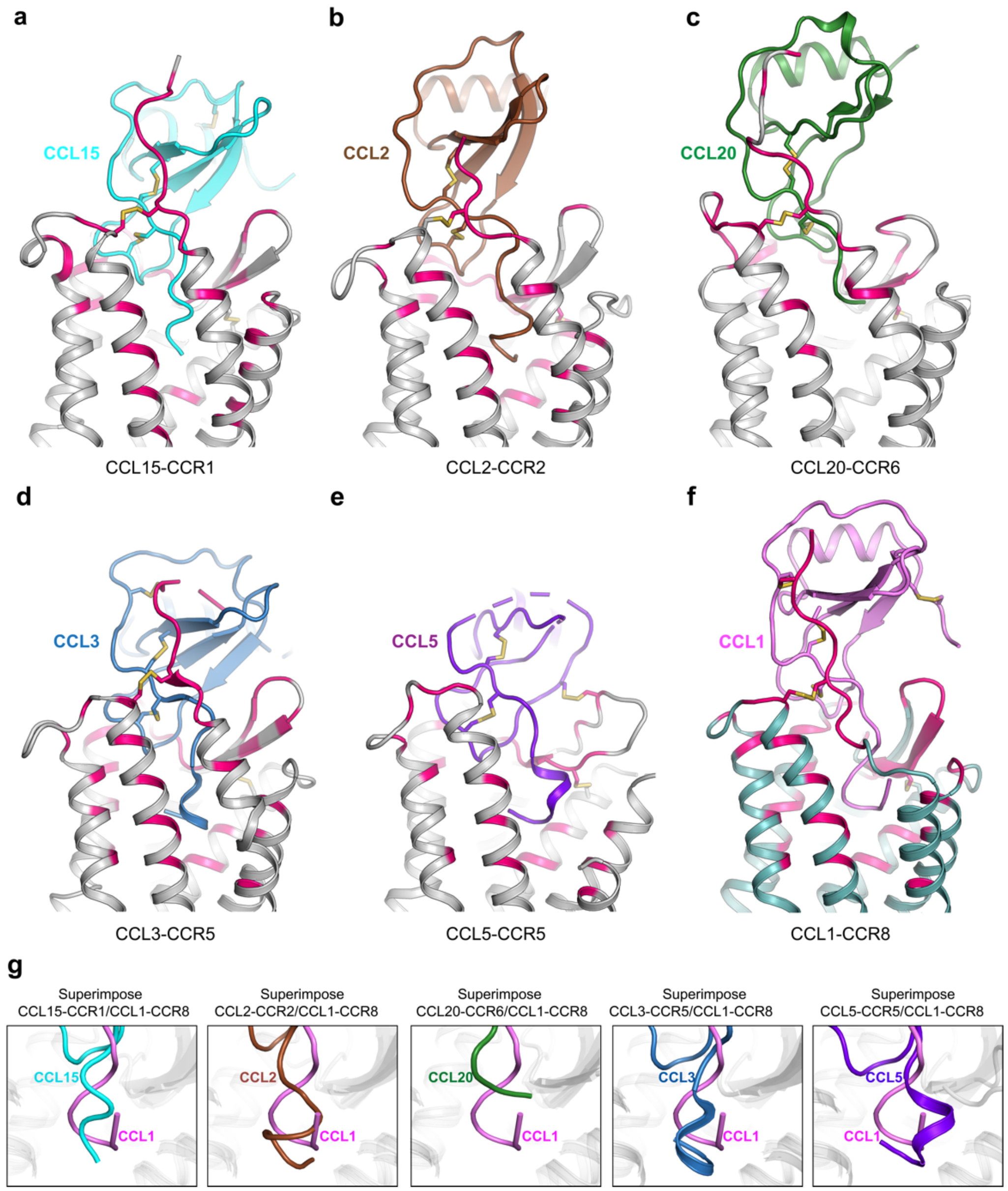

Supplementary Fig. 8: Comparison of C-C chemokine ligand - receptor ligand structures

a-f Cartoon representation of published C-C chemokine ligand - receptor structures (**a-e)**, compared to the CCL1-CCR8 structure (**f)**. All structures are aligned to the CCR8 structures, and highlight the unique N-terminal fold of CCL1, but with a similar depth of receptor core engagement as seen for other deeply engaging chemokines (CCL15, CCL2, CCL3, CCL5). The chemokines in the CCL15-CCR1-G_i (PDB ID: 7VL9), CCL2-CCR2-G_i (PDB ID: 7XA3), CCL20-CCR6-G_o (PDB ID: 6WWZ), CCL3-CCR5-G_i (PDB ID: 7F1Q), [5P7]CCL5-CCR5-G_i (PDB ID: 7F1R) and CCL1-CCR8-G_i structures are colored cyan, brown, green, blue, purple and magenta, respectively. Receptor residues within 4.5 Å of the respective chemokines are highlighted in pink. **g** Close-up overlay comparing the chemokine ligand N-terminal fold of published C-C chemokine ligand - receptor structures shown in panels **a-e** to the CCL1-CCR8 structure (magenta) shown in panel **f**.

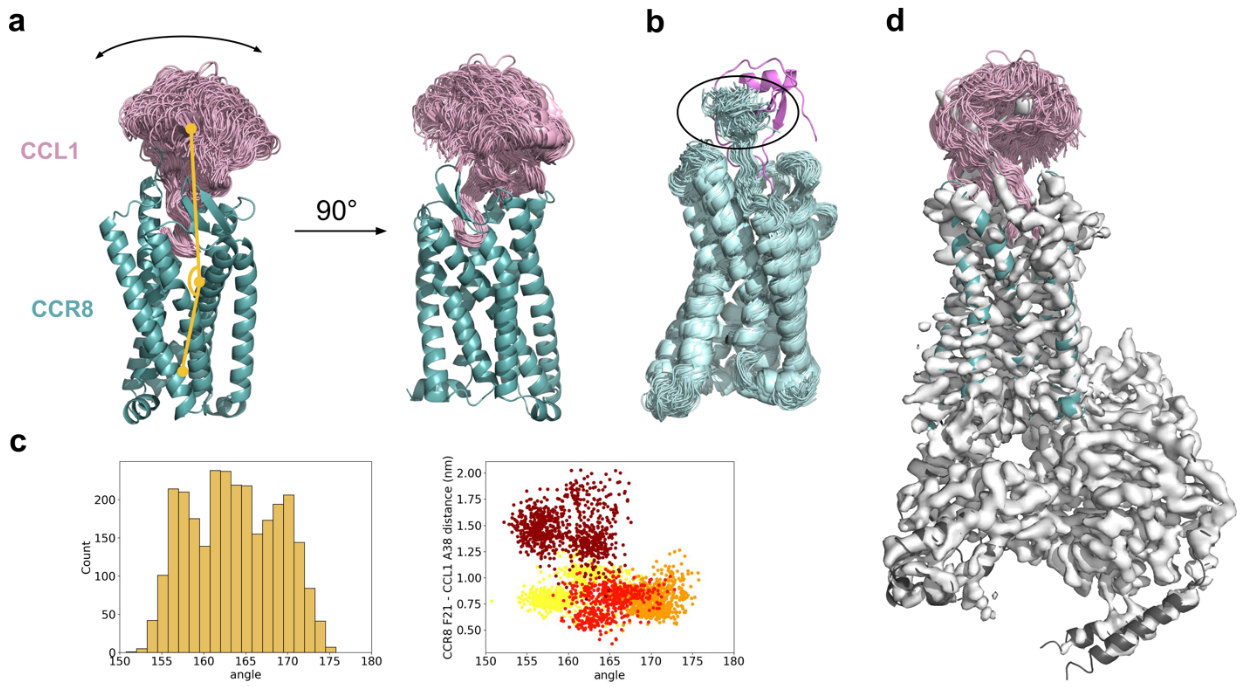

Supplementary Fig. 9: Conformational dynamics of CCL1 and CCR8 ECL3 in the CCL1-CCR8 complex

a CCL1 conformations sampled at 5 ns time intervals from four GaMD simulations of the CCL1-CCR8-G-protein complex totaling 2.8 μs simulation time show the angular distribution of the CCL1 orientation relative to CCR8. **b** Distribution of the CCR8 N-terminus at 1 ns time intervals from four GaMD simulations of the CCL1-CCR8-G-protein complex totaling 2.8 μs simulation time **c** CCL1 displays an angular distribution over four GaMD simulations of ~20 degrees, as measured by the angle between the centroid of the bottom and top halves of the CCR8 transmembrane helices and the centroid of CCL1. The angular distribution of CCL1 is independent of the CCL1 interaction with the CCR8 N-terminus, suggesting that engagement of the N-terminus of CCR8 does not influence the conformational dynamics of the CCL1 orientation. Data from the four MD runs are binned and shown in histogram format (left panel) and as individual data points with each MD run colored separately (right panel). **d** The observed conformational heterogeneity of CCL1 may explain the poor resolution of the receptor-distal CCL1 regions in our cryo-EM maps.

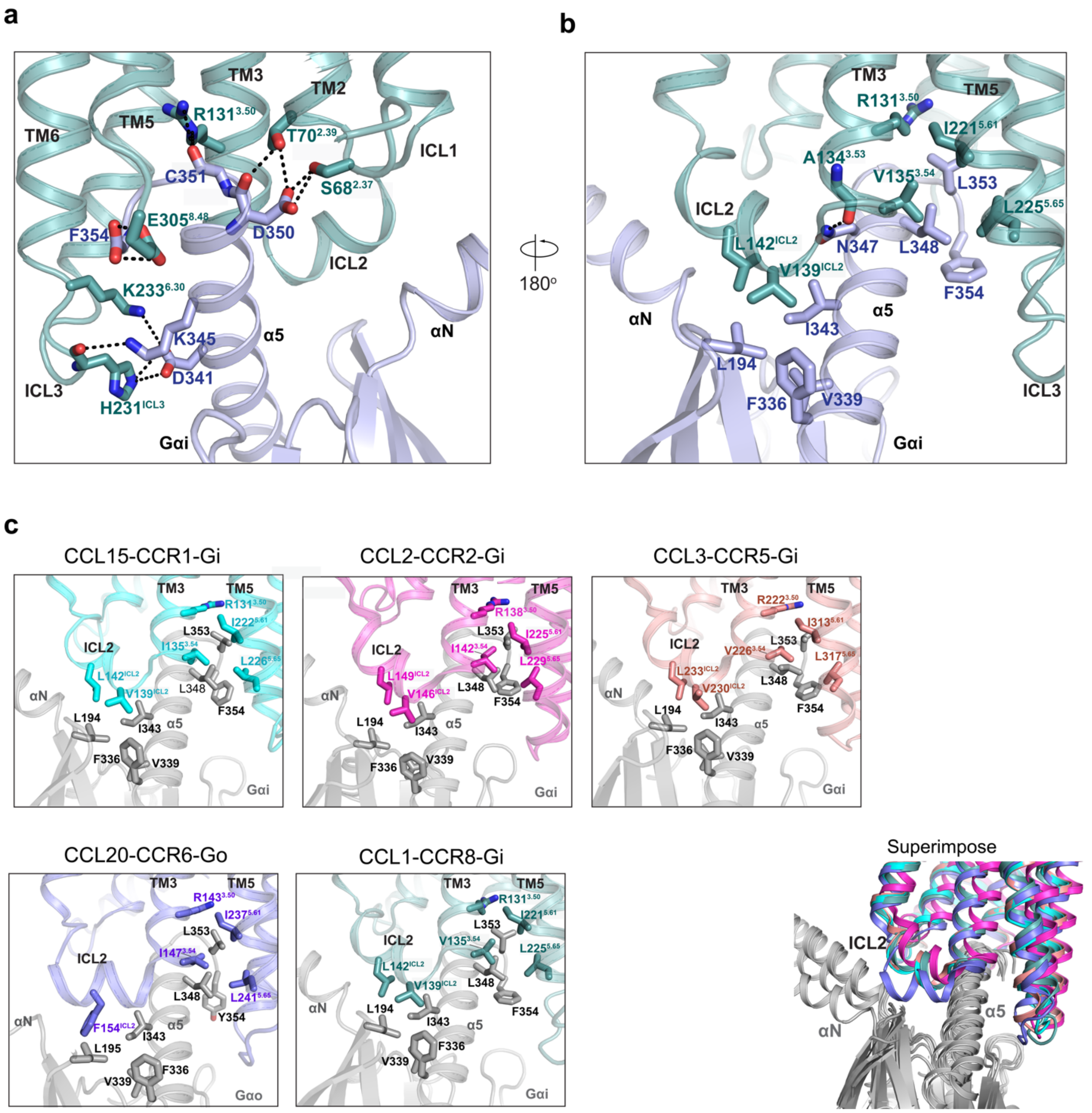

Supplementary Fig. 10: CCR8 - Gi coupling interface

a-b Binding interface between $G\alpha_i$ (blue) and CCR8 (teal). Receptor and G protein residues involved in binding are shown as sticks, with polar interactions indicated as black dashed lines. **c** Binding interface comparison between $G_{i/o}$ protein and different C-C family chemokine receptors. The receptors in the CCL15-CCR1-G_i (PDB ID: 7VL9), CCL2-CCR2-G_i (PDB ID: 7XA3), CCL3-CCR5-G_i (PDB ID: 7F1Q), CCL20-CCR6-G_o (PDB ID: 6WWZ) and CCL1-CCR8-G_i structures are colored in cyan, magenta, salmon, light blue and light teal, respectively, while the respective G proteins are colored grey. The structures are shown separately (left panels) and superimposed (right panel). Hydrophobic residues involved in the binding interface are shown as sticks.

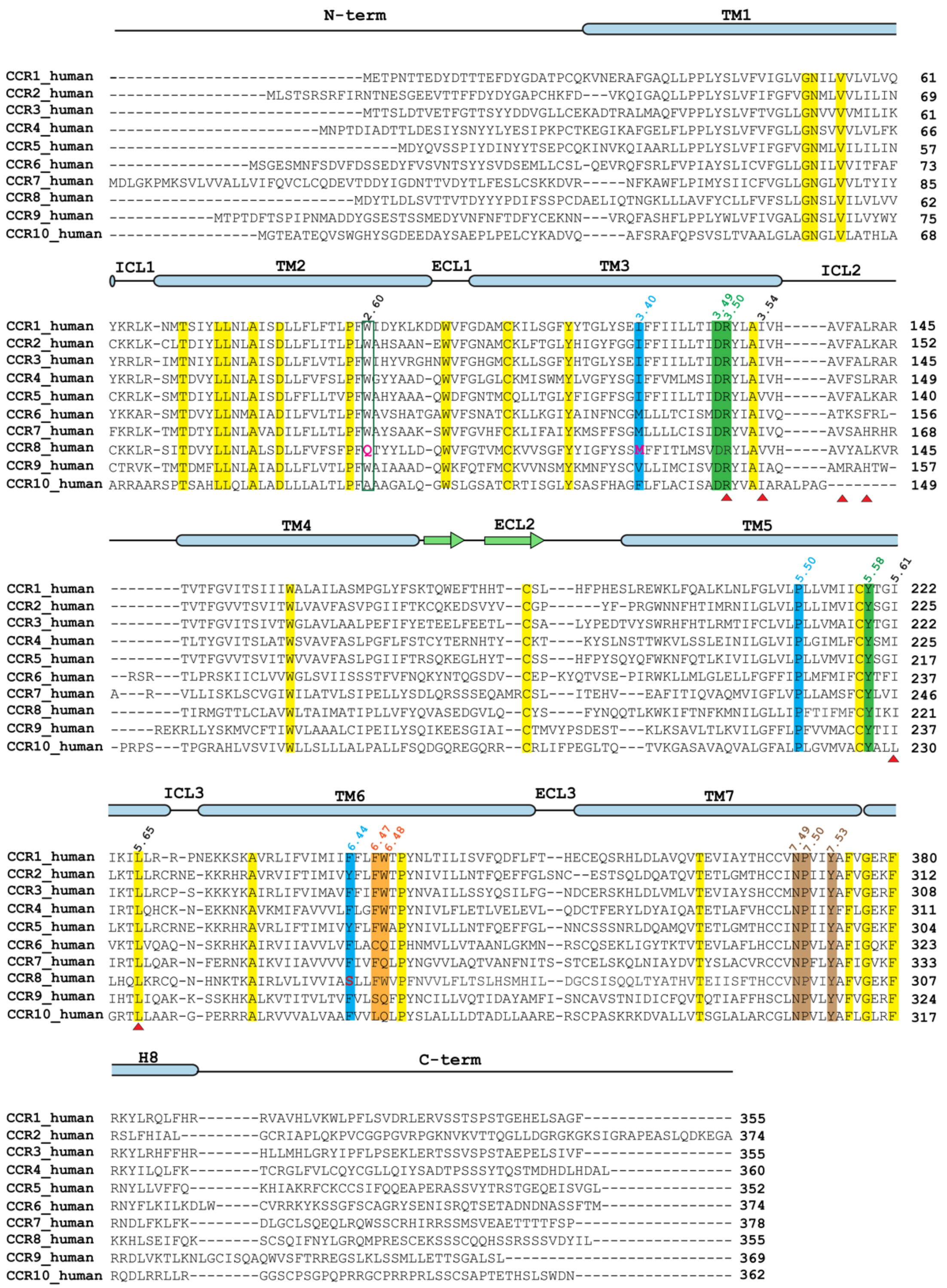

Supplementary Fig. 11: Sequence alignment of C-C family chemokine receptors.

Sequence alignment of human C-C family chemokine receptors, generated based on the GPCPdb alignment (https://gpcrdb.org/). Residues conserved across the family are highlighted in yellow. The residues corresponding to the CWxP, PIF, NPxxY and DRY motifs are highlighted in orange, blue, brown and green, respectively. The residues at position 2.60 are indicated by a green box. Hydrophobic residues involved in G protein coupling are indicated by red triangles.

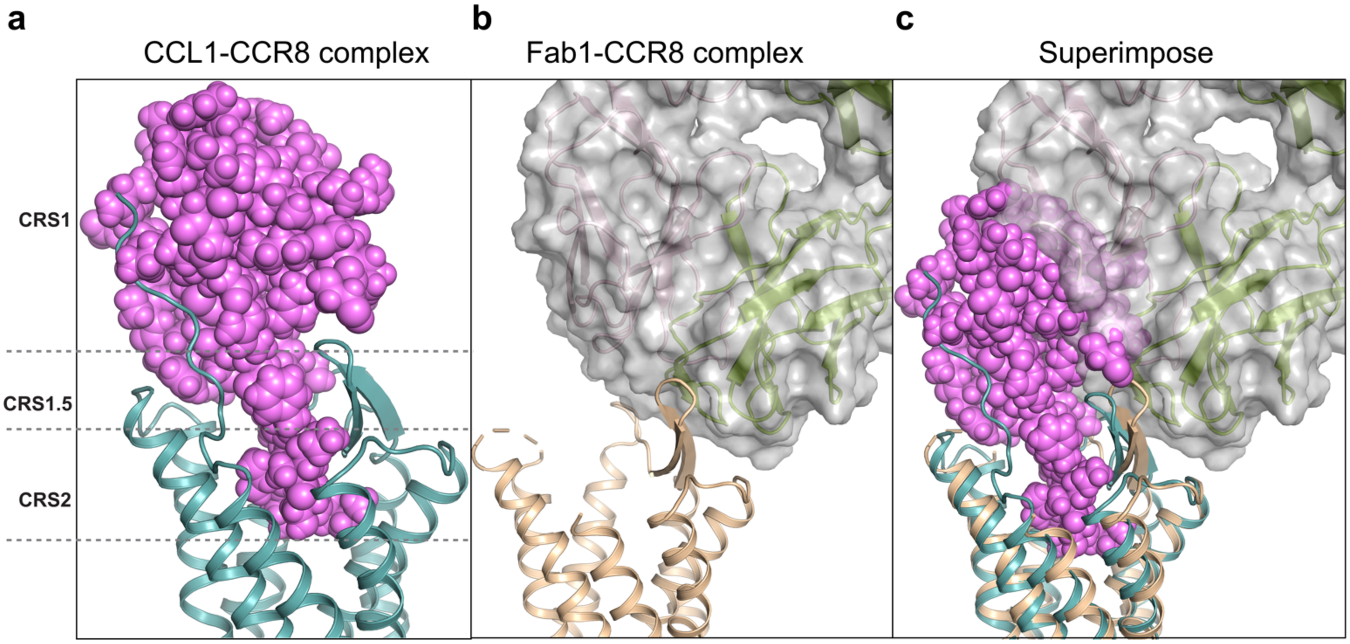

Supplementary Fig. 12: Steric clash between Fab1 and CCL1 binding to CCR8.

a-c Comparison of the CCL1 and Fab1 binding poses to CCR8, with CCL1 (colored magenta) shown as spheres bound to CCR8 (colored teal) and Fab1 displayed in cartoon (light chain colored pink and heavy chain colored green) and surface (gray) representation bound to CCR8 (colored wheat). Structures are shown separately (**a,b**) and superimposed (**c**).

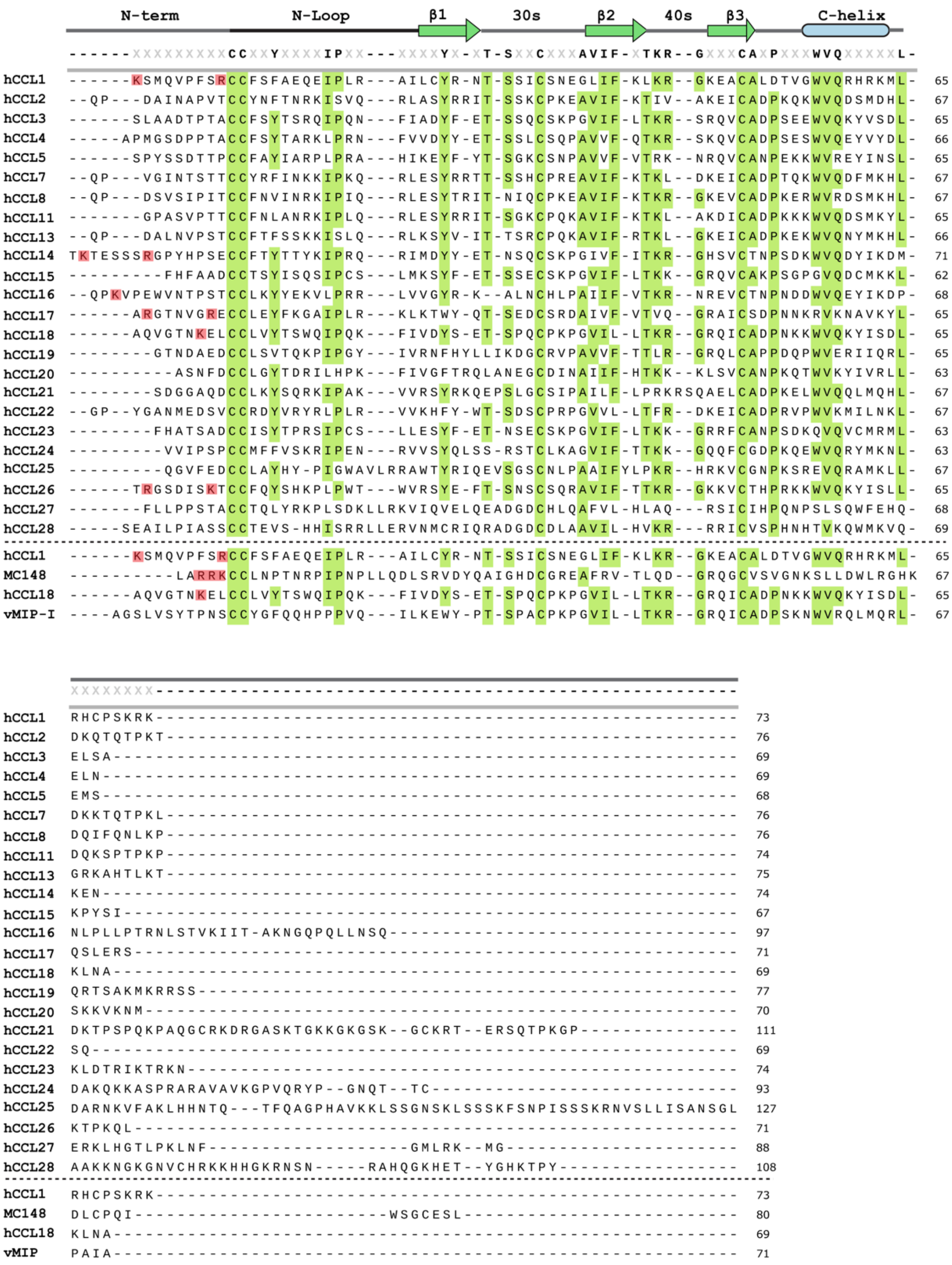

Supplementary Fig. 13: Sequence alignment of human C-C chemokines and comparison to CCR8-specific chemokines

Sequence alignment of human C-C chemokines and CCR8-specific human chemokines CCL1, CCL18 and viral chemokines MC148, vMIP. Residues with strong similar properties (consensus score higher than 50%) are highlighted in green. Lysine or arginine residues in the chemokine N-terminal sequence preceding the CC motif are colored in red. The secondary structure annotation is based on the CCL1 structure.

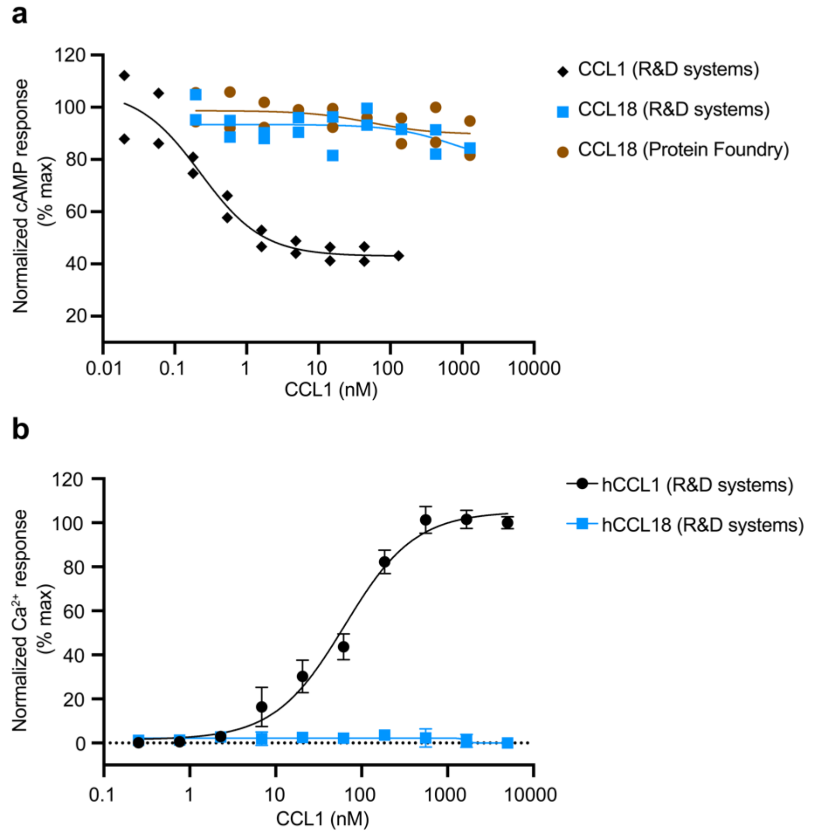

Supplementary Fig. 14: Functional assessment of CCL18 signaling through CCR8.

a cAMP inhibition measurements in HEK293T cells transiently transfected with WT hCCR8 and **b** Ca^{2+} flux measurements in a CHO.CCR8 stable cell line using the FLIPR assay in the presence of increasing concentrations of hCCL1 or hCCL18. The cAMP results were normalized as follows: 100% maximum signal, 0% media control. For the Ca^{2+} results, a negative control correction was applied and the data were normalized to the maximal hCCL1 response observed. Data are representative of two independent experiments performed in duplicates (cAMP data) or quadruplicates (Ca^{2+} data) and plotted as individual values or as mean ± standard deviation, respectively. Source data are provided as a Source Data file.

	Fab1-CCR8	CCL1-CCR8-G_i-scFv16
**PDB**	**8TLM**	**8U1U**
**EMDB**	**EMD-41470**	**EMD-41827**<br>**EMD-41828**<br>**EMD-41829**<br>**EMD-41850**
**Data Collection**		
Magnification	105,000x	165,000x
Voltage (kV)	300	300
Electron exposure (e/Å^2)	64.009	40.814
Defocus range (µm)	0.5-1.5	0.5-1.5
Pixel size (Å)	0.838	0.731
Energy filter slit width (eV)	20	20
Number of frames	60	723
**Image Processing**		
Symmetry imposed	C1	C1
Box Size (pix)	400	288
Number of micrographs (no.)	15,735	22,326
Final particle images (no.)	340,479	201,761
Map resolution (Å) overall	2.9	2.9
FSC threshold	0.143	0.143
**Refinement**		
Initial models used (PDB code)	6FG2, 5IH2, AlphaFoldDB: P51685	4OIJ, 7O7F
Map-sharpening B-factors (Å^2)	-20	N/A
Non-hydrogen atoms	5400	9507
Protein residues	696	1206
Waters	0	0
Ligands	0	1
R.m.s. deviations		
Bond lengths (Å)	0.004	0.005
Bond angles (°)	0.958	1.086
Validation		
MolProbity score	1.37	1.56
Clashscore	4.53	6.07
Poor rotamers (%)	0	0
Ramachandran Plot		
Favored (%)	97.22	96.55
Allowed (%)	2.78	3.45
Disallowed (%)	0	0

Supplementary Table 1: Cryo-EM data collection, refinement and validation statistics.

	D26A	E28A	E177A	D178A	H283A	WT
**% WT expression (mean ± SD)**	98 ± 5	86 ± 26	123 ± 16	93 ± 16	106 ± 2	100 ± 22
**Imax**	32.4	36.95	34.58	53.87	45.43	27.42
**Imax 95% CI**	28.86 to 35.83	33.13 to 40.62	31.42 to 37.65	50.94 to 56.73	41.99 to 48.71	24.35 to 30.40
**Imax fold-change over WT**	1.2	1.3	1.3	**2.0**	**1.7**	
**IC50**	0.9822	1.071	0.9341	0.4145	2.832	0.5978
**IC50 95% CI**	0.7081 to 1.366	0.7227 to 1.589	0.6820 to 1.281	0.2657 to 0.6493	1.976 to 4.035	0.4406 to 0.8133
**IC50 fold-change over WT**	1.6	1.8	1.6	0.7	**4.7**	

	Q91A	M121I	M202A	S247A	F254A	WT
**% WT expression (mean ± SD)**	110 ± 26	97 ± 15	113 ± 11	121 ± 15	104 ± 22	100 ± 26
**Imax**	76.97	N/A	55.88	27.7	46.82	33.27
**Imax 95% CI**	72.57 to 81.11		52.90 to 58.76	25.71 to 29.67	42.75 to 50.65	30.08 to 36.42
**Imax fold-change over WT**	**2.3**		**1.7**	0.8	1.4	
**IC50**	0.1081	N/A	0.3202	0.2987	1.726	0.2113
**IC50 95% CI**	0.009774 to 0.6022		0.1967 to 0.5227	0.2401 to 0.3714	1.083 to 2.755	0.1480 to 0.3010
**IC50 fold-change over WT**	0.5		1.5	1.4	**8.2**	

		F117A	Y172A	Q182A	Y184A	WT
**% WT expression (mean ± SD)**		82 ± 21	77 ± 20	104 ± 10	99 ± 11	100 ± 8
**Imax**		N/A	82.31	42	42.72	31.91
**Imax 95% CI**			79.86 to 84.56	35.31 to 48.12	35.87 to 48.48	29.54 to 34.23
**Imax fold-change over WT**			**2.6**	1.3	1.3	
**IC50**		N/A	1.333	2.077	13.62	0.2509
**IC50 95% CI**			0.4226 to 3.624	1.100 to 3.946	8.735 to 21.37	0.1879 to 0.3346
**IC50 fold-change over WT**			**5.3**	**8.3**	**54.3**	

	Y113A	Y114A	W251A	H267A	E286A	WT
**% WT expression (mean ± SD)**	65 ± 9	79 ± 16	81 ± 18	104 ± 19	130 ± 17	100 ± 29
**Imax**	N/A	74.54	N/A	46.53	74.43	27.67
**Imax 95% CI**		70.32 to 78.11		43.42 to 49.58	48.86 to 82.53	24.26 to 31.02
**Imax fold-change over WT**		**2.7**		**1.7**	**2.7**	
**IC50**	N/A	2.547	N/A	0.2684	26.05	0.4218
**IC50 95% CI**		0.9933 to 6.736		0.1779 to 0.4035	6.430 to 173.9	0.3046 to 0.5834
**IC50 fold-change over WT**		**6.0**		0.6	**61.8**	

Supplementary Table 2: Effect of CCR8 mutations on receptor expression levels and CCL1-induced CCR8 signaling activity.

Cell-based anti-Flag ELISA experiments were performed to determine the expression level of each C-terminally Flag-tagged construct transiently expressed in HEK293T cells and normalized relative to untransfected cells and a wild-type CCR8 control included in each dataset. Data are representative of two independent experiments performed in triplicates. Best-fit values and 95% confidence intervals for Imax and IC50 were calculated from the CCL1 - cAMP dose-response curves reported in **Fig. 3e & 4f** using the log(inhibitor) vs. response (three parameters) equation in Graphpad Prism v9. Data are representative of two independent experiments performed in triplicates. Mutations showing major functional effects on Imax (> 1.5-fold difference over WT) and/or IC50 (> 2-fold difference over WT) are highlighted in red. Source data are provided as a Source Data file.

Simulation box dimensions (Å)	# water molecules	Salt concentration (atom count)	Lipid composition (#, type)	Total # of atoms
127.4 x 133.7 X 180	73061	0.10 M NaCl (132 Na, 139 Cl)	476, POPC	298653

Supplementary Table 3: Molecular Dynamics system setup.

$k_{a\ step\ 1}$ ($x10^5\ M^{-1}\ s^{-1}$)	$k_{d\ step\ 1}$ ($x10^{-3}\ s^{-1}$)	$K_{D\ step\ 1}$ (nM)	$k_{a\ step\ 2}$ ($x10^{-4}\ M^{-1}\ s^{-1}$)	$k_{d\ step\ 2}$ ($x10^{-5}\ s^{-1}$)	$K_{D\ step\ 2}$ (nM)	K_D (nM)
2.44 ± 1.17	3.80 ± 2.19	~15.6	4.20 ± 1.29	2.78 ± 1.52	~0.66	1.02 ± 0.66

Supplementary Table 4: Kinetic parameters of hCCL1^{AF647} binding to CCR8.

Average on- (ka) and off-rates (kd) and affinities (K_D) for each step, as well as overall affinity, calculated for a one-to-two step fit from 3 independent LigandTracer experiments tracking hCCL1^{AF647} binding to CCR8, as shown in **Fig. 5a**. Data are presented as mean ± standard deviation. Source data are provided as a Source Data file.

CCL1 mutant	% WT activity in monocyte chemotaxis assay	% WT activity in PBMC calcium mobilization assay
WT	100	100
K24Q	2	0
S25A	20	50-60
M26A	20	10-15
Q27A	12	50-60
P29A	50	10-15
S31A	20	10-15
R32A	4	0

Supplementary Table 5: Reported CCL1 functional mutagenesis data.

CCL1 functional mutagenesis data reported in Paolini, J. F. (1995) Structural and functional analysis of the human b-chemokine I-309. [Graduate thesis] Duke University, with CCL1 mutants showing the most dramatic loss in activity compared to WT highlighted in yellow.

Uncropped scans of Western Blots related to Figure 1c and Supplementary Figure 2e

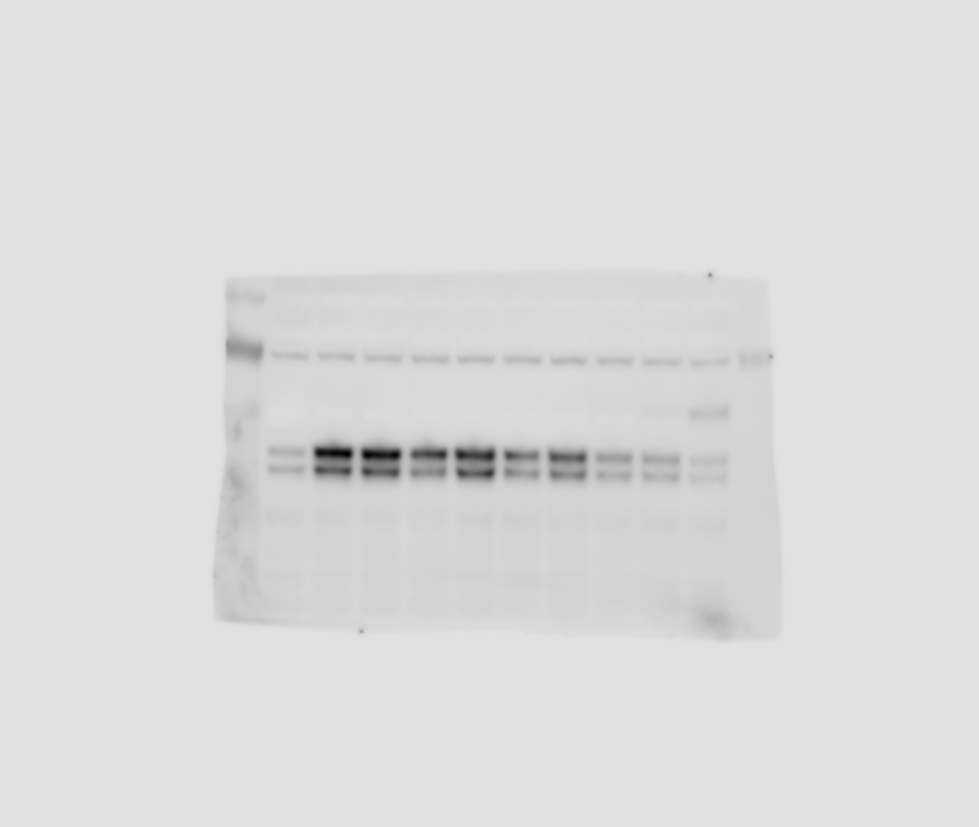

Uncropped anti- phosphoERK Western Blot

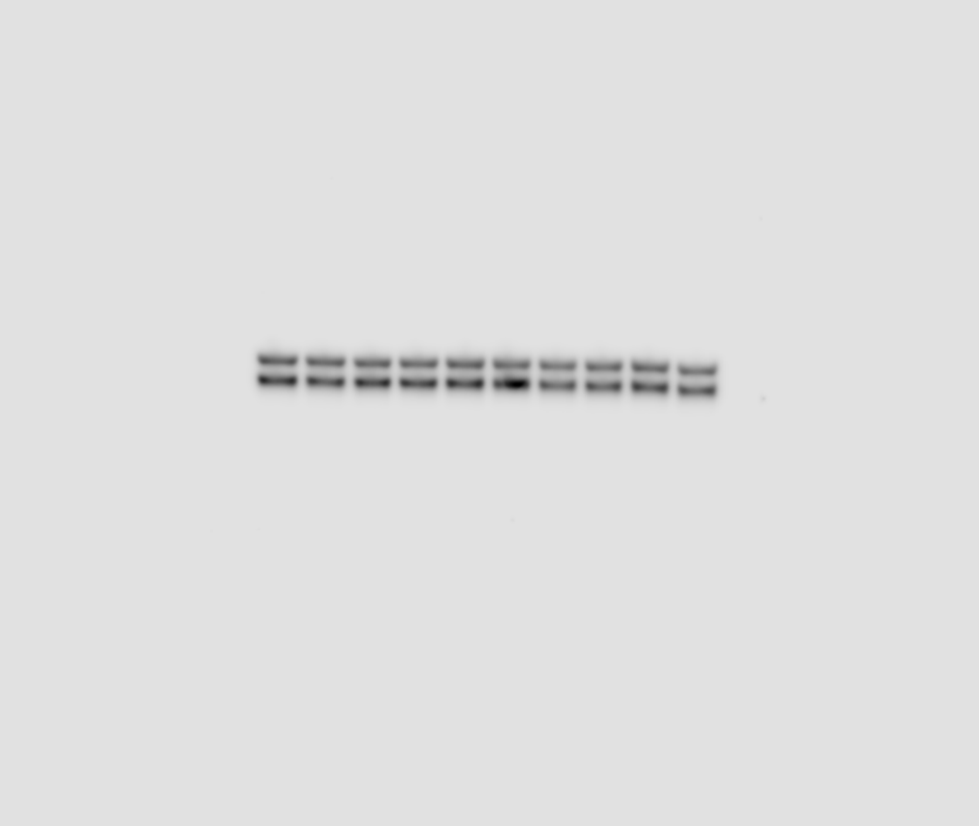

Uncropped anti- ERK Western Blot
