# Supplementary material for: Structural basis of antibody inhibition and chemokine activation of the human CC chemokine receptor 8
Source: Nat Commun. 2023 Dec 1;14:7940. doi: 10.1038/s41467-023-43601-8 (PMC10692165; doi:10.1038/s41467-023-43601-8)
Supplement: Supplementary file 3 — Reporting Summary [file 41467_2023_43601_MOESM3_ESM.pdf]

nature portfolio

Corresponding author(s): Sascha Rutz, James T. Koerber, Matthieu Masureel

Last updated by author(s): Oct 26, 2023

Reporting Summary

Nature Portfolio wishes to improve the reproducibility of the work that we publish. This form provides structure for consistency and transparency in reporting. For further information on Nature Portfolio policies, see our Editorial Policies and the Editorial Policy Checklist.

Statistics

For all statistical analyses, confirm that the following items are present in the figure legend, table legend, main text, or Methods section.

n/a	Confirmed	
☐	☒	The exact sample size (*n*) for each experimental group/condition, given as a discrete number and unit of measurement
☐	☒	A statement on whether measurements were taken from distinct samples or whether the same sample was measured repeatedly
☒	☐	The statistical test(s) used AND whether they are one- or two-sided<br>*Only common tests should be described solely by name; describe more complex techniques in the Methods section.*
☒	☐	A description of all covariates tested
☒	☐	A description of any assumptions or corrections, such as tests of normality and adjustment for multiple comparisons
☐	☒	A full description of the statistical parameters including central tendency (e.g. means) or other basic estimates (e.g. regression coefficient) AND variation (e.g. standard deviation) or associated estimates of uncertainty (e.g. confidence intervals)
☒	☐	For null hypothesis testing, the test statistic (e.g. *F*, *t*, *r*) with confidence intervals, effect sizes, degrees of freedom and *P* value noted<br>*Give P values as exact values whenever suitable.*
☒	☐	For Bayesian analysis, information on the choice of priors and Markov chain Monte Carlo settings
☒	☐	For hierarchical and complex designs, identification of the appropriate level for tests and full reporting of outcomes
☒	☐	Estimates of effect sizes (e.g. Cohen's *d*, Pearson's *r*), indicating how they were calculated

Our web collection on statistics for biologists contains articles on many of the points above.

Software and code

Policy information about availability of computer code

Data collection	EPU 2.3.0.79REL
Data analysis	TraceDrawer 1.9.2, Graphpad Prism 9.5.0, UCSF Chimera, 1.14, UCSF ChimeraX, 1.5, cryoSPARC, 3.3.1, Pymol, 2.5.4, Coot, 0.9.8.4, Phenix & embedded MolProbity, 1.19.1-4122-final, FlowJo 10.5.3 & 10.6.1, Image Studio Lite 5.2.5

For manuscripts utilizing custom algorithms or software that are central to the research but not yet described in published literature, software must be made available to editors and reviewers. We strongly encourage code deposition in a community repository (e.g. GitHub). See the Nature Portfolio guidelines for submitting code & software for further information.

Data

Policy information about availability of data

All manuscripts must include a data availability statement. This statement should provide the following information, where applicable:

- Accession codes, unique identifiers, or web links for publicly available datasets
- A description of any restrictions on data availability
- For clinical datasets or third party data, please ensure that the statement adheres to our policy

Sequence information of CCR8 and CCL1 was obtained from the Uniprot database (CCR8: P51685, CCL1: P22362). Source data is provided for Fig. 1a-c, Fig. 3e-f, Fig. 4f-g, Fig. 5 a-b and Supplementary Fig. 2c,e. The 3D cryo-EM density maps of Fab1-CCR8 and CCL1-CCR8-Gi-scFv16 have been deposited into the Electron Microscopy Data Bank [https://www.ebi.ac.uk/pdbe/emdb] under accession codes EMD-41470 (Fab1-CCR8), EM-41827(Gi-scFv16), EM-41828(CCL1-CCR8),

EM-41829 (composite map of CCL1-CCR8-Gi-scFv16) and EM-41850 (consensus map of CCL1-CCR8-Gi-scFv16). The coordinates of the Fab1-CCR8 and CCL1-CCR8-Gi-scFv16 complexes have been deposited in the PDB [https://www.rcsb.org] under accession codes 8TLM and 8U1U, respectively. Source data are provided with this paper. Initial coordinate and simulation input files as well as a coordinate files of the final output of the GaMD simulations have been deposited in an open public repository [https://zenodo.org/records/10038937].

Research involving human participants, their data, or biological material

Policy information about studies with human participants or human data. See also policy information about sex, gender (identity/presentation), and sexual orientation and race, ethnicity and racism.

Reporting on sex and gender	N/A
Reporting on race, ethnicity, or other socially relevant groupings	N/A
Population characteristics	N/A
Recruitment	N/A
Ethics oversight	N/A

Note that full information on the approval of the study protocol must also be provided in the manuscript.

Field-specific reporting

Please select the one below that is the best fit for your research. If you are not sure, read the appropriate sections before making your selection.

☒ Life sciences ☐ Behavioural & social sciences ☐ Ecological, evolutionary & environmental sciences

For a reference copy of the document with all sections, see nature.com/documents/nr-reporting-summary-flat.pdf

Life sciences study design

All studies must disclose on these points even when the disclosure is negative.

Sample size	Sample sizes were not predetermined for this study. Sample size for cryo-EM data were determined by the availability of microscope time. All cell-based assays were performed at a minimum in independent duplicates, as further detailed in the methods section. The sample size for the cell-based assays was chosen based on the level of variability across samples and taking into consideration the time-sensitive experimental set up.
Data exclusions	No data were excluded from the analysis.
Replication	Cell-based measurements were performed in duplicate or triplicate, as detailed in respective methods sections. Molecular Dynamics simulations were performed in quadruplicate. All attempts at replication were successful.
Randomization	Randomization is not applicable for the experiments involving cryo-EM, as the data were collected automatically and did not involve choosing. For cell-based assays data was recorded in varying order.
Blinding	Blinding is not necessary or valid for structural determination, as for the cryoEM datasets imaging data were collected automatically. For the cell-based assays, blinding was not necessary since the experiments are quantitative in nature.

Reporting for specific materials, systems and methods

We require information from authors about some types of materials, experimental systems and methods used in many studies. Here, indicate whether each material, system or method listed is relevant to your study. If you are not sure if a list item applies to your research, read the appropriate section before selecting a response.

Materials & experimental systems

n/a	Involved in the study
☐	☒ Antibodies
☐	☒ Eukaryotic cell lines
☒	☐ Palaeontology and archaeology
☐	☒ Animals and other organisms
☒	☐ Clinical data
☒	☐ Dual use research of concern
☒	☐ Plants

Methods

n/a	Involved in the study
☒	☐ ChIP-seq
☐	☒ Flow cytometry
☒	☐ MRI-based neuroimaging

Antibodies

Antibodies used

[Antibody, Clone, Supplier, Catalog #]

anti-human CCR8 mAb1, produced at Genentech
anti-gD isotype control, produced at Genentech
anti-human OX40, 3C8, produced at Genentech
anti-human ERBB2, 4D5, produced at Genentech
anti-hIgG, produced at Genentech
anti-human CD45 BV510, HI30, BD Biosciences, Catalog # 563204
anti-human CD3 BUV395, SK7, BD Biosciences, Catalog # 564001
anti-human CD8 FITC, RPA-T8, BD Biosciences, Catalog # 561948
anti-human CD14 PerCP-Cy5.5, 63D3, Biolegend, Catalog # 367110
anti-human CD4 BV421, RPA-T4, Biolegend, Catalog # 300532
anti-human FOXP3 PE, 236A/E7, ThermoFisher Scientific, Catalog # 12-4777-42
rabbit anti-Flag pAb, XXX, Sigma-Aldrich, XXX
AF647-anti-hIgG, Jackson ImmunoResearch Laboratories, Catalog # 109-606-170
AF647-anti-RbIgG, Jackson ImmunoResearch Laboratories, XXX
AF647-conjugated AffiniPure F(ab')2 Fragment Goat anti-Human IgG, Fcγ fragment specific; Jackson ImmunoResearch; Catalog # 109-606-006
anti-human CCR8 APC, 433H, BD Biosciences, Catalog # 566897
mouse monoclonal anti-FLAG M2-FITC antibody, Sigma Aldrich, Catalog # F4049
phospho-p44/42 MAPK (ERK1/2) (Thr202/Tyr204); D13.14.4E; Cell Signaling Technology, Catalog # 4370S
p44/42 MAPK (ERK1/2); 137F5; Cell Signaling Technology, Catalog # 4695S
anti-human GAPDH, Bio-Rad, AbD22549, Catalog # HCA272

Validation

Validation of the anti-human CCR8 mAb1 antibody was performed in this study through biochemistry analysis, cryo-EM 3D reconstruction and cell-based assays as described. In-house produced antibodies were validated to bind their respective target by SPR. Validation of commercial antibodies used in this study were done by the manufacturer and details of these validation efforts are described on the manufacturer's website as detailed below.

anti-human CD45 BV510, HI30, BD Biosciences
https://www.bdbiosciences.com/en-us/products/reagents/flow-cytometry-reagents/research-reagents/single-color-antibodies-ruo/bv510-mouse-anti-human-cd45.563204
anti-human CD3 BUV395 (SK7, BD Biosciences)
https://www.bdbiosciences.com/en-us/products/reagents/flow-cytometry-reagents/research-reagents/single-color-antibodies-ruo/buv395-mouse-anti-human-cd3.564001
anti-human CD8 FITC (RPA-T8, BD Biosciences)
https://www.bdbiosciences.com/en-us/products/reagents/flow-cytometry-reagents/research-reagents/single-color-antibodies-ruo/fitc-mouse-anti-human-cd8.561948
anti-CD14 PerCP-Cy5.5 (63D3, Biolegend)
https://www.biolegend.com/nl-be/products/percp-cyanine5-5-anti-human-cd14-antibody-12802?GroupID=BLG14394
anti-CD4 BV421 (RPA-T4, BioLegend)
https://www.biolegend.com/de-at/cell-health/brilliant-violet-421-anti-human-cd4-antibody-7151#:~:text=The%20RPA%2DT4%20antibody%20binds,binding%20and%20inhibit%20syncytia%20formation.
anti-FOXP3 PE (236A/E7, ThermoFisher Scientific)
https://www.thermofisher.com/antibody/product/FOXP3-Antibody-clone-236A-E7-Monoclonal/12-4777-42
anti-CCR8 APC (433H, BD Biosciences)
https://www.bdbiosciences.com/en-us/products/reagents/flow-cytometry-reagents/research-reagents/single-color-antibodies-ruo/apc-mouse-anti-human-ccr8-cd198.566897
anti-FLAG M2-FITC antibody (F4049, Sigma Aldrich)
https://www.sigmaaldrich.com/US/en/product/sigma/f4049
anti-phospho-p44/42 MAPK (ERK1/2) (Thr202/Tyr204) (D13.14.4E, Cell Signaling Technology)
https://www.cellsignal.com/products/primary-antibodies/phospho-p44-42-mapk-erk1-2-thr202-tyr204-d13-14-4e-xp-rabbit-mab/4370
anti-p44/42 MAPK (ERK1/2) (137F5, Cell Signaling Technology)
https://www.cellsignal.com/products/primary-antibodies/p44-42-mapk-erk1-2-137f5-rabbit-mab/4695
anti-human GAPDH (AbD22549, Bio-Rad)
https://www.bio-rad-antibodies.com/monoclonal/human-gapdh-antibody-abd22549-higg1-hca272.html?f=purified

Eukaryotic cell lines

Policy information about cell lines and Sex and Gender in Research

Cell line source(s)	HEK293 (human embryonic kidney cells, Expi293F Invitrogen)<br>CHO (Chinese Hamster Ovary Cell, CHO-K1 ATCC, CCL-61)<br>DTCs (human colorectal dissociated tumor cells, Discovery Life Sciences)<br>PBMCs (human peripheral blood mononuclear cells, Genentech blood donor program)<br>CHO.hCCR8 cells (Chinese Hamster Ovary Cell, CHO-K1 ATCC, CCL-61, modified at Genentech to express hCCR8)<br>HuCCR8.Jurkat cells (Jurkat (Clone E6-1), ATCC, TIB-152, modified at Genentech to express hCCR8)
Authentication	Cell lines were not authenticated.
Mycoplasma contamination	Each cell line tested negative for mycoplasma contamination.
Commonly misidentified lines<br>(See ICLAC register)	No commonly misidentified lines were used.

Animals and other research organisms

Policy information about studies involving animals; ARRIVE guidelines recommended for reporting animal research, and Sex and Gender in Research

Laboratory animals	New Zealand White (NZW) rabbits (Charles River Laboratories, Hollister, CA), female, 6 months old.
Wild animals	The study did not involve wild animals.
Reporting on sex	All rabbits were female.
Field-collected samples	No field-collected samples were used in this study.
Ethics oversight	Animals used in these studies were maintained in an Association for Assessment and Accreditation of Laboratory Animal Care (AAALAC)-accredited animal facility. All experiments were performed in compliance with Genentech's Institutional Animal Care and Use Committee (IACUC) and National Institutes of Health's Office of Laboratory Animal Welfare Guidelines. Approval of the study design was obtained from the Genentech IACUC prior to the start of this work.

Note that full information on the approval of the study protocol must also be provided in the manuscript.

Plants

Seed stocks	N/A
Novel plant genotypes	N/A
Authentication	N/A

Flow Cytometry

Plots

Confirm that:

☒ The axis labels state the marker and fluorochrome used (e.g. CD4-FITC).

☒ The axis scales are clearly visible. Include numbers along axes only for bottom left plot of group (a 'group' is an analysis of identical markers).

☒ All plots are contour plots with outliers or pseudocolor plots.

☒ A numerical value for number of cells or percentage (with statistics) is provided.

Methodology

Sample preparation	Human PBMCs were isolated by Ficoll gradient centrifugation from buffy coats from healthy donors, collected as part of the Genentech blood donor program with written informed consent, and approval from the Western Institutional Review Board.

	Cells were stained with eFluor 780-conjugated Fixable Viability Dye (ThermoFisher Scientific) and 2 ug/mL mAb1, anti-OX40 (positive control), anti-ERBB2 (negative control), or anti-hIgG (negative control) for 20 min at 4°C followed by secondary detection with AF647-conjugated AffiniPure F(ab')2 Fragment Goat anti-Human IgG, Fcγ fragment specific (Jackson ImmunoResearch) for 10 min at 4°C. To test the commercial antibody (Clone 433H, BD Biosciences), cells were surface stained with eFluor 780-conjugated Fixable Viability Dye and APC-conjugated anti-Human CCR8 at 1 mL per test. Cells were then intracellularly stained using the eBioscience Foxp3/Transcription Factor Staining Buffer Set (ThermoFisher Scientific) according to the manufacturer's protocol. Antibodies used to define T cell populations were CD45 (HI30), CD3 (SK7), CD8 (RPA-T8), and CD14 (63D3) from BD Biosciences, CD4 (RPA-T4) from BioLegend, and FOXP3 (236A/E7) from ThermoFisher Scientific. Flow cytometry was performed on a Fortessa X-20 (BD Biosciences) and analyzed with FlowJo software (BD Biosciences, Version 10.5.3).
Instrument	Fortessa X-20 (BD Biosciences); FACSCelestaTM Cell Analyzer (BD Biosciences)
Software	FlowJo Version 10.5.3 & 10.6.1 (BD Biosciences)
Cell population abundance	Does not apply
Gating strategy	Cell were stained with fluorescently labeled antibodies and flow cytometry was performed as described in the methods section. The gating strategy is outlined in Suppl. Fig. 1. Briefly, singlets were identified by FSC-H/FSC-A and SSC-W/SSC-A gating, immune cells were gated based on FCS-A/SSC-A followed by gating on CD45 positive and FVD negative cells. T cells were further identified based on CD3 expression and further subset into CD8 positive/CD4 negative T cells, CD4 positive/Foxp3 negative T cells and CD4 positive/Foxp3 positive Treg cells.

☒ Tick this box to confirm that a figure exemplifying the gating strategy is provided in the Supplementary Information.
